# Quantitative Determination of Lethal Toxin Proteins in Culture Supernatant of Human Live Anthrax Vaccine *Bacillus anthracis* A16R

**DOI:** 10.3390/toxins8030056

**Published:** 2016-02-25

**Authors:** Xiaodong Zai, Jun Zhang, Ju Liu, Jie Liu, Liangliang Li, Ying Yin, Ling Fu, Junjie Xu, Wei Chen

**Affiliations:** Laboratory of Vaccine and Antibody Engineering, Beijing Institute of Biotechnology, Beijing 100071, China; zaixiaodong@163.com (X.Z.); justforhere@126.com (J.Z.); 18701522421@163.com (Ju.L.); liujieer2015@163.com (Ji.L.); byebyelll@163.com (L.L.); yinying1028@sina.cn (Y.Y.); fuling3436@163.com (L.F.)

**Keywords:** *Bacillus anthracis*, anthrax toxin, lethal toxin, protective antigen, lethal factor, anthrax vaccine, A16R, quantitative determination

## Abstract

*Bacillus anthracis* (*B. anthracis*) is the etiological agent of anthrax affecting both humans and animals. Anthrax toxin (AT) plays a major role in pathogenesis. It includes lethal toxin (LT) and edema toxin (ET), which are formed by the combination of protective antigen (PA) and lethal factor (LF) or edema factor (EF), respectively. The currently used human anthrax vaccine in China utilizes live-attenuated *B. anthracis* spores (A16R; pXO1+, pXO2−) that produce anthrax toxin but cannot produce the capsule. Anthrax toxins, especially LT, have key effects on both the immunogenicity and toxicity of human anthrax vaccines. Thus, determining quantities and biological activities of LT proteins expressed by the A16R strain is meaningful. Here, we explored LT expression patterns of the A16R strain in culture conditions using another vaccine strain Sterne as a control. We developed a sandwich ELISA and cytotoxicity-based method for quantitative detection of PA and LF. Expression and degradation of LT proteins were observed in culture supernatants over time. Additionally, LT proteins expressed by the A16R and Sterne strains were found to be monomeric and showed cytotoxic activity, which may be the main reason for side effects of live anthrax vaccines. Our work facilitates the characterization of anthrax vaccines components and establishment of a quality control standard for vaccine production which may ultimately help to ensure the efficacy and safety of the human anthrax vaccine A16R.

## 1. Introduction

*Bacillus anthracis* (*B. anthracis*), the etiological agent causing anthrax, is a Gram-positive, spore-forming bacterium. It can be used in biowarfare or bioterror attacks and has been a top bioterrorism concern since the 2001 anthrax attacks in the USA [1,2]. Fully virulent forms of *B. anthracis* carry two large plasmids: pXO1 and pXO2. The pXO1 plasmid encodes anthrax toxins, and pXO2 encodes proteins that form the poly-d-glutamic acid capsule. Anthrax toxin (AT), including lethal toxin (LT) and edema toxin (ET), are A− type exotoxins each composed of two proteins. The A component is either the lethal factor (LF, 89 kDa) or edema factor (EF, 90 kDa), and the B component is the protective antigen (PA, 83 kDa) [3]. LF is a zinc metalloprotease that inactivates mitogen-activated protein kinase kinases (MAPKK). EF is a calmodulin-dependent adenylyl cyclase that elevates intracellular cAMP levels by converting ATP to cAMP. Meanwhile, PA is a non-toxic cell-binding component responsible for transporting LF and EF into the cell, where they exert their toxic effects [4]. LT is the major virulence factor of *B. anthracis*, which causes a range of effects on cellular functions and plays essential roles during multiple steps of the disease [3,5,6,7]. It not only directly causes tissue damage and lethality at late stages of infection but also plays essential roles in impairing the host innate immune response at the initial stage of infection to ensure a systemic infection [8,9,10,11,12].

The threat of anthrax biowarfare has stimulated the development of vaccines for mass scale immunization. Almost all anthrax vaccines in use or development rely on PA as the primary immunogen [2]. The United States (Anthrax Vaccine Adsorbed; AVA) and United Kingdom (Anthrax Vaccine Precipitated; AVP) have used component vaccines that are based on culture filtrates of avirulent *B. anthracis* strains, V770-NP1-R strain and Sterne strain 34F2, respectively [2]. Meanwhile, China has utilized toxin-producing live-attenuated *B. anthracis* spores (A16R; pXO1+, pXO2−) as a human anthrax vaccine [13]. More defined and recombinant PA (rPA)-based anthrax vaccines are under development currently [14,15,16,17]. These approved anthrax vaccine strains produce large amounts of PA, which plays an important role in immunity and prophylaxis against anthrax. Various studies carried out with anthrax vaccines in different animal models indicate the relevance of PA as a key component of the vaccine [16,18]. Antibodies generated against PA, especially those that have anthrax toxin neutralization activity, have been established as being critical for immunity to anthrax [19,20,21].

Evidence also exists to support that LF evokes a more rapid and stronger host immune response in comparison to the other two anthrax toxins, PA and EF [22,23]. Vaccination studies have indicated that not only PA, but also LF, is capable of conferring protective immunity [24,25,26]. Following immunization with either AVA or AVP, individuals have shown antibody responses to both PA and LF [27,28,29]. Meanwhile, both PA and LF specific antibodies have been detected in sera taken from naturally infected anthrax patients [27]. The presence of an antibody response to LF was reported to enhance the protection afforded by anthrax vaccines in animals against a spore challenge [30,31]. To summarize, the two components of LT proteins, PA and LF, play important roles in determining the immune responses to anthrax vaccines.

Although the efficacy and safety of all these anthrax vaccines have been established, concerns over their relatively high rate of side effects remain [32]. The inoculation of anthrax vaccines is known to cause a number of local and systemic reactions. These side effects may be caused by the AT and especially the major virulence factor LT. Therefore, LT proteins have key effects on both the immunogenicity and toxicity of anthrax vaccines. Component vaccines are based on culture filtrates containing LT proteins expressed by vaccine strains. For live spore vaccines, the vaccine strains may produce a large amount of LT proteins *in vivo* after immunization. These LT proteins of both component vaccines and live spore vaccines may result in intense side effects. Therefore, determining the quantity and biological activity of LT proteins expressed by anthrax vaccine strains is a meaningful endeavor.

Prior studies have evaluated the expression of toxins in culture of some anthrax vaccine strains [33,34,35,36]. Moreover, the quantification of LT proteins in serum has been used for diagnostics and evaluation of medical countermeasures [37,38,39,40,41,42]. However, the toxin expression patterns of vaccine A16R in culture conditions are still unclear. In this study, we explored the LT protein expression patterns of the A16R strain throughout various stages in the growth process using the Sterne strain (another widely-used anthrax vaccine) as a control. We developed a sandwich ELISA and cytotoxicity-based method for the quantitative detection of LT proteins (PA and LF) in the culture supernatants of the A16R and Sterne strains. Our work may help to improve the understanding of the expression patterns of LT proteins in the culture process. Since the expression of LT proteins in an *in vitro* culture system may also have some significance related to the expression *in vivo*, the findings can be helpful in the evaluation of LT protein expression after vaccine inoculation. It can also be used in quality control during production of the live-attenuated anthrax vaccine *B. anthracis* A16R and ultimately help to ensure the efficacy and safety of human anthrax vaccines.

## 2. Results

### 2.1. Expression and Degradation of LT Proteins in Culture Supernatant Detected by Western Blot

**Figure 1:**
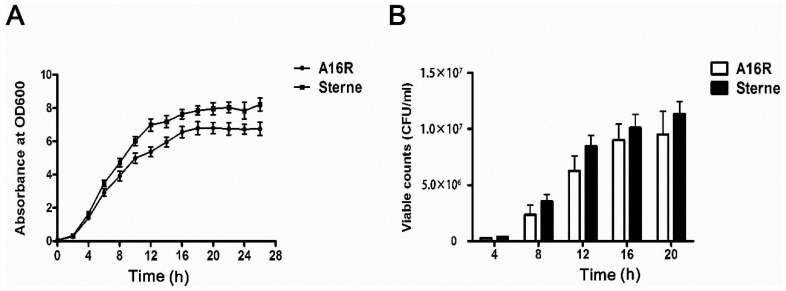
Growth curves (**A**) and viable counts (**B**) of *B. anthracis* A16R and Sterne throughout the bacterial growth phase. The optical density (OD_600_) of culture was determined every 2 h, and the viable counts were performed every 4 h. Data are presented as the mean ± standard error (SEM) from three independent experiments.

*B. anthracis* A16R and Sterne are both live-attenuated anthrax vaccines that are used widely [2]. Since LT proteins have key effects on both the immunogenicity and toxicity of anthrax vaccines, quantitatively determining the LT proteins expressed by A16R and Sterne is useful. Here, we explored expression patterns of LT proteins of the A16R and Sterne strains throughout the various stages and conditions of *in vitro* culture for 28 h. Analysis of the growth kinetics showed a sigmoidal profile that reached the late exponential phase after about 12 h; meanwhile, the viable cell count reached approximately 10^7^ viable cells per mL (Figure 1A,B) and then peaked with the onset of the stationary phase. Similar patterns of growth were observed with the *B. anthracis* A16R and Sterne strains. However, the growth of *B. anthracis* A16R was slightly slower compared with that of the Sterne strain (Figure 1A). In addition, throughout the growth phase, viable counts of the Sterne strain were similar but slightly higher than that of the A16R strain (Figure 1B), which may influence the production of LT proteins in the culture.

**Figure 2:**
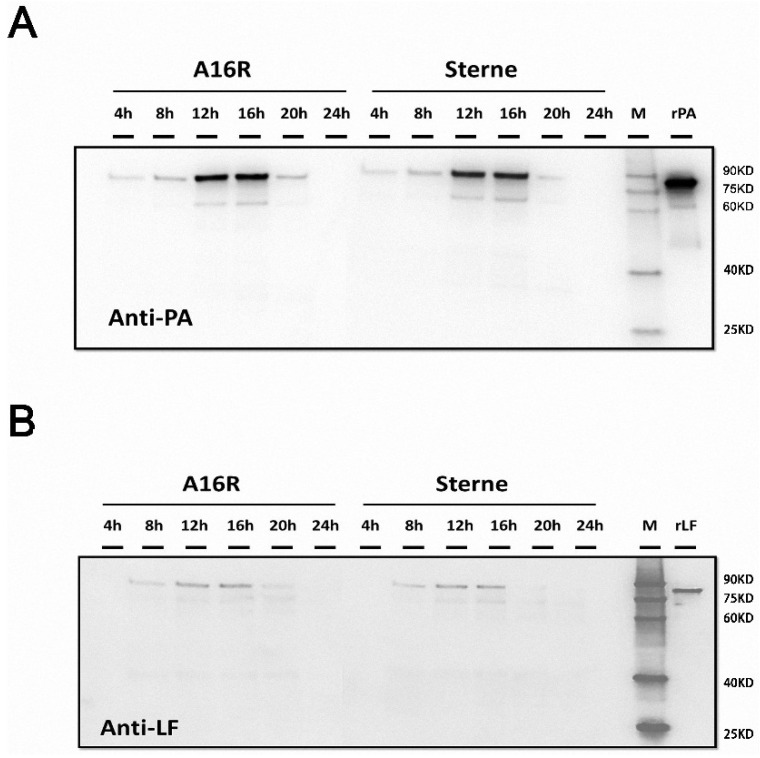
Western blot analysis of *B. anthracis* A16R and Sterne culture supernatants. (**A**,**B**) Time course samples at 4, 8, 12, 16, 20 and 24 h. Twenty microliters of culture supernatant was loaded for each sample. rPA (50 ng)/recombinant LF (rLF) (10 ng) was also loaded as a positive control. The Western blot membrane was incubated with a 1:1000 dilution of anti-PA or anti-LF rabbit polyclonal antibodies (pAbs).

After obtaining the growth characteristics of the strains, we evaluated the expression of LT proteins. After cultivation and sampling, filtered supernatants were analyzed by Western blotting, and the proteins were visualized and recorded using image analysis. The expression and degradation of LT proteins (PA and LF) in the culture supernatants were analyzed by Western blotting (Figure 2). Throughout the culture period, the amount of LT proteins increased in the logarithmic phase at first and then began to decrease after the stabilization period. While a number of smaller bands were present, the band corresponding to the full-length antigen decreased. Earlier samples in the time course showed relatively more abundant full-length antigens by Western blotting, with increased degradation observed at later points. For the A16R and Sterne strains, both PA and LF proteins showed degradation over the culture period. According to the Western blot results, LF seemed to be subject to slightly less degradation compared to PA. Thus, the stability of PA and LF in the culture supernatant appeared to differ.

In the classical model of anthrax toxin production, PA is produced as PA83 which specifically binds to cell surface receptors TEM8 and CMG2. PA83 is then cleaved by a cell surface protease (furin) into PA63 and a 20 kDa fragment. PA63 spontaneously forms heptamers, which recruit LF and EF, completing the formation of the anthrax toxin [43]. Only intact PA and LF have biological activities. After degradation, PA and LF cannot assemble a toxin complex and will have lost their biological activities. Therefore, while both intact and degraded LT proteins can be detected in the culture supernatants, only a portion of them would be active.

### 2.2. A16R and Sterne Strains Show Similar LT Protein Expression Patterns in Culture, with a Greater Extent of Degradation of PA than LF, as Quantified by ELISA and a Cytotoxicity-Based Method

As the Western blot analysis showed the expression and degradation of LT proteins in the culture supernatants qualitatively, we subsequently carried out quantitative detection of these proteins to determine the precise expression patterns of anthrax vaccine strains A16R and Sterne cultured *in vitro*. Antigen levels during the manufacturing fermentation process should be detected for PA and LF using sensitive and specific methods. Therefore, we established two quantitative methods, ELISA and a cytotoxicity-based method, to detect the LT proteins in the culture supernatant.

ELISA is a commonly used method for quantitative detection of PA and LF [34,44]. pAbs specific for PA (or LF) were coated on the plates, and then diluted culture supernatant samples containing PA (or LF) were added, followed by the HRP-labeled anti-PA (or LF) monoclonal antibody (mAb). We have validated that the epitope of the anti-PA mAb is the PA domain 4 (596–735), and the epitope of the anti-LF mAb is the LF central region (276–576). Therefore, these two mAbs can be used to capture not only full-length LT but also partially degraded LT. Although some partially degraded LT fragments may not be recognized using these antibodies if they have lost the specific epitopes, the pattern of LT degradation can still be discerned through the detection of full-length LT and partially degraded LT.

**Figure 3:**
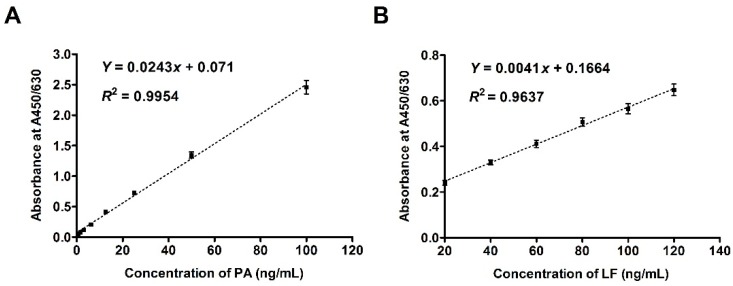
Standard curves for sandwich ELISA to determine PA and LF concentrations. (**A**) Standard curve of PA; (**B**) Standard curve of LF. The standards rPA and rLF were added to the plates at the starting concentrations of 100 ng/mL and 120 ng/mL in 100 μL per well, respectively. Data are presented as the mean ± SEM from three independent experiments.

After establishing the standard curves for the sandwich ELISA (Figure 3), the concentrations of PA and LF could be quantitatively determined. We defined the limit of detection (LOD) as the lowest concentration of LT proteins in the samples that could be measured with a signal to the background signal (S/N) ratio of 3/1(S/N). The LOD of ELISA for PA was 2 ng/mL, and the linear dynamic range was 3.1–100 ng/mL. While the LOD of ELISA for LF was 5 ng/mL, and the linear dynamic range was 20–120 ng/mL. From the standard curve (Figure 3), we concluded that the ELISA method to quantitate LT proteins is efficient.

**Figure 4:**
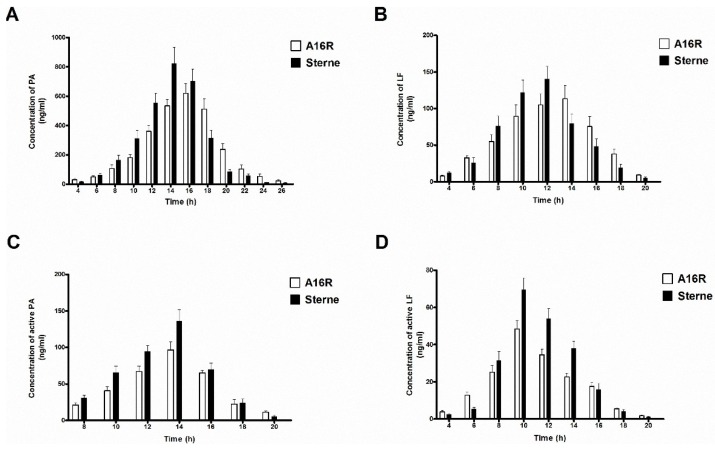
PA and LF levels in samples of filter-sterilized culture supernatants from cultures of *B. anthracis* A16R and Sterne. (**A**) PA and (**B**) LF levels detected by sandwich ELISA; (**C**) PA and (**D**) LF levels detected by a cytotoxicity-based method. Data are presented as the mean ± SEM from three independent experiments.

The LT proteins PA and LF present in the culture supernatants were first determined using antigen-capture ELISA (Figure 4A,B), and the A16R and Sterne strains showed similar expression patterns. Throughout the culture growth process, the amount of LT component proteins PA and LF increased in the logarithmic phase at first and then began to decrease after a stabilization period. In the beginning of the culture period, the expression rates of LT proteins of the A16R and Sterne strains were greater than those of their degradation, and therefore the concentrations of PA and LF were increasing. As the growth of the strains continued, increasingly more PA and LF proteins degraded, resulting in the reduction of their concentrations. The highest PA and LF levels in the culture supernatant were approximately 600–900 ng/mL and 100–150 ng/mL, respectively, between 12 and 16 h. The LT expression of the Sterne strain was slightly higher than that of the A16R strain, which may be the cause of the different growth patterns. The Sterne strain showed a more rapid growth profile and higher viable cell counts in the stabilization period.

The ratio of PA:LF in the samples was approximately 5:1 throughout the cultivation period. This finding actually confirmed several previous reports of *in vitro* toxin production (PA:LF:EF, 20:5:1) [37] and AVP manufacture (PA:LF, 4:1) [34], as well as measurements in another *in vivo* study of Ames spore infection in rabbits [44,45]. Meanwhile, production levels of PA and LF detected in culture of the UK-licensed AVP were ~7.89 μg/mL and 1.85 μg/mL, respectively. Moreover, the amounts of LT proteins expressed by anthrax strains in the culture supernatant differed, which may be attributed to the different culture conditions used in these studies.

Over the course of the culture growth process, the degradation of PA and LF increased. The ELISA method detects the total concentrations of PA and LF, including the degraded proteins, which would not accurately reflect the functionally active LT components. Since the mouse macrophage cell line J774 is uniquely sensitive to LT and can be used in detection of LT lethal activity [46], we used these cells in a cytotoxicity-based assay to measure the concentrations of active PA and LF proteins. In this experiment, the active PA (PA83) specifically bound to cell surface receptors TEM8 and CMG2. It was then cleaved by a cell surface protease (furin) into PA63 and a 20 kDa fragment. PA63 would have spontaneously formed heptamers, which recruited LF. As the cell surface receptors available for binding PA were highly abundant, adding excess PA (1 µg/mL) or LF (50 ng/mL) could allow better quantitation of the amount of active LF or PA.

With a fixed amount of excess PA (or LF), any change in cytotoxicity would be attributed to the varying amounts of LF (or PA). We used two of the same samples to detect active PA and LF individually. The active LF in samples was assessed based on the ability, together with excess added PA, to kill J774 cells *in vitro*. Using the same approach, the active PA in samples was also assessed.

As described above, excess PA (1 μg/mL) and varying amounts of 10× culture supernatant samples were applied, and cell viability was then assessed 4 h later by the MTT assay. As the concentration of PA was in excess, the decisive factor was the concentration of LF, which could be obtained based on the cell viability. Thus, a cytotoxicity-based method for detection of active LF was developed. This method could be used also in the quantitative detection of active PA.

Results shown in Figure 4C,D were similar to the expression patterns detected by ELISA. In the culture supernatants of the A16R and Sterne strains, active PA and LF showed an increase at the beginning and then a decrease. The highest active PA and LF levels in the culture supernatant were approximately 100–150 ng/mL and 50–80 ng/mL, respectively, at the stabilization period (10–14 h). Levels of active PA and LF of the Sterne strain were slightly higher than those of the A16R strain. Different from the previous PA:LF ratio determined by ELISA above, the ratio of active PA to active LF was about 2:1. These results provide the first precise determination of the proportion of active LT proteins in culture supernatant of the A16R strain.

**Figure 5:**
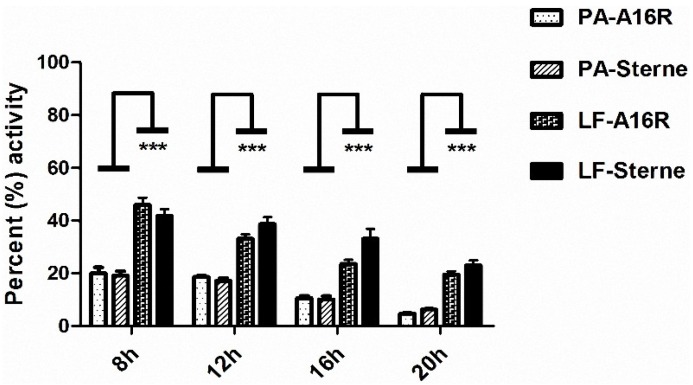
Percentages of active PA and LF in *B. anthracis* A16R and Sterne culture supernatants. The percentages of active PA and LF in the culture supernatants at time points 8, 12, 16 and 20 h were calculated. Data are presented as the mean ± SEM from three independent experiments. Statistical significance was determined by unpaired two-tailed t test. *** *p* ≤ 0.001.

By using an ELISA and a cytotoxicity-based method, we successfully quantitated the amounts of LT toxin components PA and LF throughout the culture growth process, including levels of the total proteins and those of the active components. As the total PA and LF proteins degrade, they lose their activity. Therefore, calculating the percentages of PA and LF activity in *B. anthracis* A16R and Sterne culture supernatants would allow us to explore the differences in their stability. Intense degradation of the LT proteins was observed. The percentage of PA activity decreased from 19.6% to 5.5%, while that of LF decreased from 43.9% to 21.3% during the culture process. Especially in the later stages in the fermentation, most of the LT proteins were degraded (Figure 5). The extent of degradation of PA was greater than that of LF in the culture supernatant, as reported in previous studies [34].

### 2.3. Protease Inhibitors Can Reduce the Protease Activity in Culture Supernatants and Maintain a High Level of LT Proteins in the Stabilization Period.

**Figure 6:**
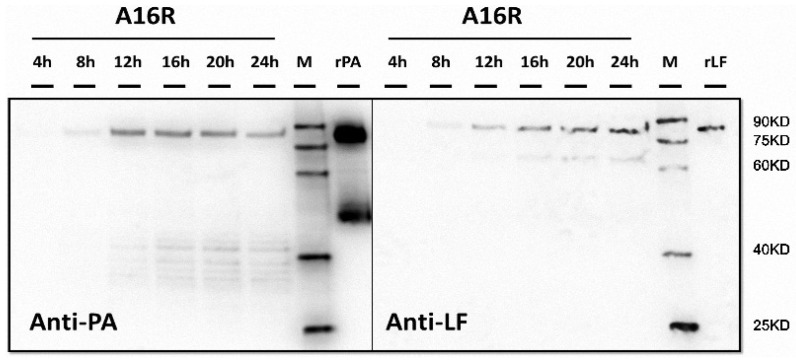
Western blot analysis of samples of filter-sterilized culture supernatants from cultures of *B. anthracis* A16R with protease inhibitors. Time course samples at 4, 8, 12, 16, 20 and 24 h. Twenty microliters of culture supernatant was loaded for each sample. rPA (100 ng)/rLF (10 ng) was also loaded as a positive control. The Western blot membrane was incubated with a 1:1000 dilution of anti-PA or anti-LF rabbit pAb.

As we have mentioned, over the course of the culture growth process, the level of LT proteins increased in the logarithmic phase at first and then began to decrease after a stabilization period. The protease activity in culture supernatants may be the main reason for this decrease. Therefore, we used Western blot and ELISA to detect the expression of LT in the presence of protease inhibitors during the A16R culture process. The Western blot results showed that the amount of LT (PA and LF) during the A16R culture growth process increased in the logarithmic phase (4–12 h) at first and then maintained a high level in the stabilization period (12–24 h) (Figure 6).

**Figure 7:**
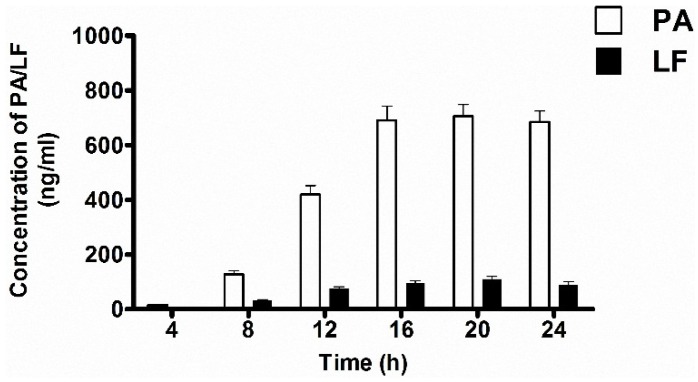
PA and LF levels in samples of filter-sterilized culture supernatants from cultures of *B. anthracis* A16R with protease inhibitors. PA and LF levels were detected by sandwich ELISA. Time course samples at 4, 8, 12, 16, 20 and 24 h. Data are presented as the mean ± SEM from three independent experiments.

It was also validated by the quantitative determination of PA and LF (Figure 7). The highest PA and LF levels in the culture supernatant were approximately 650–750 ng/mL and 80–120 ng/mL, respectively, and maintained this high level in the stabilization period between 12–24 h. The highest LT proteins levels of the A16R culture with protease inhibitors was slightly larger than those without inhibitors. The ratio of PA:LF in the samples was approximately 6:1 throughout the cultivation period.

Therefore, we concluded that through the entire culture period, LT expression and degradation both occurred. During the initial stage, LT levels increased since its expression was faster than its rate of degradation. However, in the stationary phase, with the accumulation of proteases, the degradation rate of LT may have been more rapid than its expression. The LT level then rapidly declined in the stabilization phase. After adding protease inhibitor in the culture to reduce the protease activity, the LT degradation was inhibited. Consequently, the amount of LT gradually increased and maintained a high level stably.

### 2.4. LT Proteins are All Monomeric in Culture Supernatants and Show Lethal Activity as Determined by a Macrophage Cell Lysis Assay

After the quantitative detection of LT proteins in culture supernatant, we confirmed the presence of active PA and active LF. During cellular intoxication, PA83 binds to cell surface receptors CMG2 and TEM8, releasing its 20-kDa amino-terminus, leaving PA63 bound to the cell surface [4]. PA63 forms heptameric and octameric complexes, which are capable of binding LF. The PA63-LF complexes can translocate LF into the cell cytoplasm [4,43,47]. Within the cell, LF, a zinc-dependent endoproteinase, exerts its lethal effects.

Some studies have shown that an alternative mechanism for LT assembly may exist [43]. Such a pathway would be independent of the anthrax toxin receptors and the cell surface protease. A specific protease may be present in the serum that is capable of cleaving PA83 to PA63, which then spontaneously forms heptamers. The heptamers would recruit LF, resulting in pre-formed LT in the bloodstream. Therefore, we performed a co-immunoprecipitation (co-IP) assay to detect any pre-formed LT potentially present in the culture supernatants. In the experiment designed to quantify pre-formed LT, an anti-PA pAb was used to first capture PA and an anti-LF mAb to detect LF. The reverse co-IP assay was performed as well, in which an anti-LF pAb was used to pull down LF, and an anti-PA mAb was used for detection.

After epitope analysis, we found that the specific binding epitopes of the anti-PA and LF mAbs are located externally on the LT heptamer complex. We also detected the rPA, rLF and LT heptamer complexes at fixed amounts in the co-IP assay as a control. The co-IP assay results showed that both methods could detect the presence of the LT heptamer complexes in the control. If any PA83 proteins in the samples were cleaved to PA63, the latter would then spontaneously form heptamers and in turn recruit LF, resulting in pre-formed LT heptamer complexes. These complexes then could be detected by the co-IP assay and the reverse pull-down assay in this experiment. However, neither method within their detection range could identify the presence of pre-formed LT heptamer complexes in culture supernatants from 4 to 24 h (Table 1). Thus, LT proteins were determined to be all monomeric in the culture supernatants.

**Figure 8:**
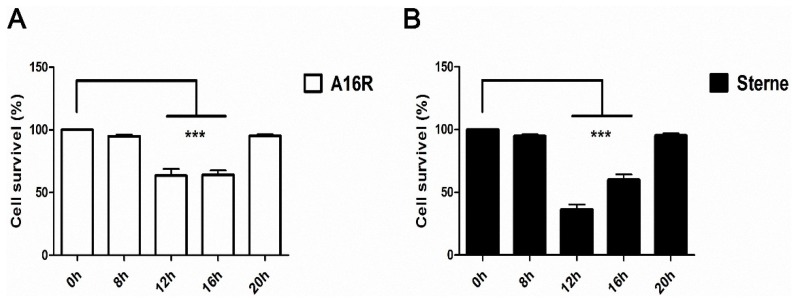
Macrophage cell lysis assay of LT lethal activity in *B. anthracis* A16R and Sterne culture supernatants. (**A**) Lethal activity of A16R culture supernatant; (**B**) Lethal activity of Sterne culture supernatant. Samples were measured at 0, 8, 12, 16 and 20 h. The growth medium was removed by cautious aspiration and replaced with 100 μL of new medium containing culture supernatant samples (dilution 1:10). Data are presented as the mean ± SEM from three independent experiments. Statistical significance was determined by unpaired two-tailed t test. *** *p* ≤ 0.001.

Thus, we explored the biological activities of the culture supernatants with both monomeric PA and monomeric LF that can form the LT complexes on the cell surface. The lethal activity of LT proteins in the culture supernatants throughout the time course was determined in a macrophage cell lysis assay. The results showed that the culture supernatant samples from 8–12 h had the highest lethal toxin activity (Figure 8). The cytotoxic activity of the Sterne strain culture supernatant was somewhat stronger than that of the A16R culture supernatant, due to its higher concentration of active PA and active LF. Taken together with the Western blot data, these observations confirmed that some of the LF and PA proteins in the culture supernatants remained intact in the early stages, although most of them were degraded and had lost functionality at later stages in the fermentation process. The loss of function meant that the degraded proteins failed to bind to the macrophage cell surface as active LT to be internalized to kill the cells. Overall, results of the *in vitro* culture experiments suggested that after vaccination the *B. anthracis* strains could express large amounts of LT proteins with lethal activity.

## 3. Discussion

Anthrax caused by *B. anthracis* is an important endemic disease of public health concern. As the management of anthrax remains a top priority among biowarfare/bioterror agents, the effectiveness and safety of anthrax vaccines must be improved. The A16R strain is the only licensed human live anthrax vaccine in China and plays an important role in anthrax prevention and control. LT proteins play a critical role in the pathogenesis of anthrax disease and have key effects on the immunogenicity and toxicity of the anthrax vaccine. Thus, the quantitative determination and analysis of the biological activity of lethal toxin proteins expressed by A16R are meaningful. Here, we explored expression patterns of the A16R strain LT proteins throughout the various stages in the growth process with the Sterne strain as a control.

This study clearly demonstrated the presence of significant amounts of LF in the culture supernatants of the A16R and Sterne strains. LT proteins in particular influence the efficacy of anthrax vaccines, since they have been proposed to affect humoral immunity. Antibody responses to LF may enhance the protection offered by anthrax vaccines. Compared to the currently used anthrax vaccine AVA that contains only low variable amounts of LF, the A16R and AVP vaccines contain significant amounts of LF. Moreover, the lethal activity of LT proteins throughout the time course was determined. The lethal activity of culture supernatant of the Sterne strain was somewhat stronger than that of A16R, due to its higher concentration of active PA and active LF. While the *in vitro* experiments cannot absolutely reflect the situation *in vivo*, they may have some correlative aspects. Our analysis of the anthrax LT expression in an *in vitro* culture system may also have some significance related to the expression *in vivo*. Our findings can be helpful in the evaluation of LT protein expression *in vivo* after vaccine inoculation. In other words, investigating the toxin expression levels of the A16R and Sterne strains *in vitro* may inform us on the efficacy and safety of these vaccines.

The highest PA and LF levels in the culture supernatants of the A16R and Sterne strains were much lower than those of AVP. The different expression levels of LT proteins in the culture supernatant may be caused by the different culture conditions. In fact, the experimental conditions in our work were quite different from those of the UK-licensed AVP. As the production conditions of AVP uses specific medium components and a different culture process, a direct comparison of its toxin expression levels with those of vaccine strains in this study is not meaningful. Nevertheless, the quantitative detection of LT proteins can be used as a quality control indicator for the production of the anthrax vaccine A16R. We will attempt to establish a quality control standard using the concentration of LT proteins so that only vaccine strains that express LT proteins stably within the standard range can be used in anthrax vaccine A16R production. Such a standard will ensure the stability of vaccine strains. Thus, our results may ultimately help to ensure the efficacy and safety of human anthrax vaccines. We selected the most common *in vitro* culture conditions in this study that would allow for toxin determination and for quality control standards of vaccine strains to be quickly and easily established. We also selected Sterne as a control. Thus, the comparative analysis of A16R with other strains can be obtained under the same culture conditions.

In earlier studies of *B. anthracis* grown statically, levels of PA peaked at around 38 h and then declined [34], while little or no degradation of antigens in the UK-licensed AVP culture supernatant was observed [35]. In this study, the amounts of LT proteins PA and LF increased in the logarithmic phase at first and then began to decrease after the stabilization period. The protease activity in culture supernatants may be the main reason for this decrease. The protease inhibitors can reduce the protease activity in culture supernatants and maintain a high level of LT proteins in the stabilization period.

One interesting finding is the pattern of degradation of active PA and LF to non-active forms. The biological effects of LT proteins in culture supernatant are due to the activity of the main components PA and LF. We explored the differences in degradation and stability between PA and LF through calculating their relative activity levels. These results provide the first precise determination of the proportions of active LT proteins in culture supernatant. The analysis showed that the percentages of PA and LF activity decreased significantly in the later stages, when most of the LT proteins had degraded. PA was degraded more extensively than LF, which suggests that PA is the limiting factor for LT activity in the culture supernatants.

We also found that LT proteins were all monomeric in the culture supernatants since the co-IP assay (or the reverse pull-down assay) could not detect the existence of pre-formed LT heptamer complexes. We concluded that both full-length PA83 and non-specific degraded PA, but not PA63, were present in the culture supernatants. Since the cleavage of PA83 to PA63 can occur only by the activity of a protease (furin) at the cell surface, no PA63 heptamers complexes could be detected in the culture supernatants. This finding may be attributed to differences between conditions in the bloodstream and the *in vitro* culture which would not have the specific protease capable of cleaving PA83 to PA63.

**Figure 9:**
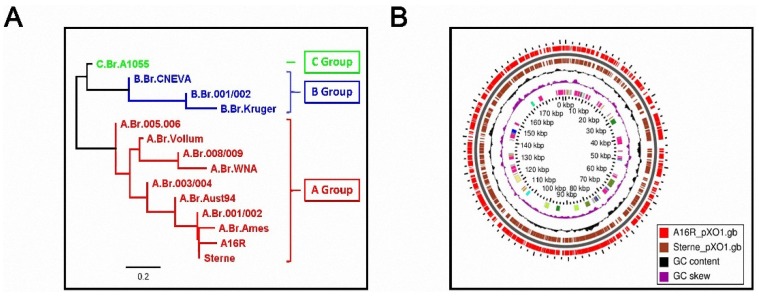
Relationship between A16R and Sterne strains. (**A**) Sub-lineages of several *B. anthracis* isolates by canonical SNP analysis; (**B**) Basic local alignment search tool (BLAST) atlas genome map between A16R pXO1 and Sterne pXO1. From the inside: purple and black bars denote GC skew and GC content, respectively. Brown bars indicate the coding DNA sequence (CDS) regions in the Sterne strain, and whole red bars indicate regions on the A16R genome where a BLAST hit occurred within Sterne.

In this study, we found that the A16R and Sterne strains showed similar growth curves and viable counts, as well as similar patterns of PA and LF production. The results suggested that the PA and LF production from A16R was comparable to that of other vaccine strains. The main reason for this similarity in toxin expression may be the level of homology, especially in pXO1, between the A16R and Sterne strains. Canonical single nucleotide polymorphism (SNP) analysis of several *B. anthracis* isolates showed that the A16R and Sterne strains belong to the same sub-lineages (Figure 9) [48,49]. The genome map comparing A16R pXO1 (Genbank: NZ_CP001975) and Sterne pXO1 (Genbank: NZ_CP009540) also confirmed the similarities between them [50].

In conclusion, we have provided general baseline data profiling the kinetics of growth and production of PA and LF, as well as the relationship between PA and LF degradation in human anthrax vaccines. We developed a sandwich ELISA and cytotoxicity-based method for the quantitative detection of LT proteins PA and LF. The A16R and Sterne strains showed similar LT protein expression patterns *in vitro*, with a greater extent of degradation of PA than LF, as quantified by ELISA and a cytotoxicity-based method. Furthermore, we found that protease inhibitors could reduce the protease activity in culture supernatants and maintain a high level of LT proteins in the stabilization period. Finally, we examined the lethal activity of the culture supernatants over a time course and demonstrated that the LT proteins expressed by A16R and Sterne were all monomeric and cytotoxic, which may be the main reason for the side effects of anthrax vaccines. Only limited studies have been performed on the growth and toxin expression patterns of *B. anthracis* strains under culture conditions. Our report is the first detailed study focused on the quantitative detection of LT proteins in the culture supernatant of the A16R strain. These findings improve our understanding of the vaccine strain A16R and may ultimately help to ensure the efficacy and safety of human anthrax vaccines A16R.

## 4. Experimental Section

### 4.1. Reagents

The recombinant proteins rPA and rLF used in this study were purified from *Escherichia coli* as described previously [46,51]. Rabbit pAbs to PA and LF were produced by inoculation of rabbit and then purified from serum. Mouse mAb to PA and LF were prepared by fusing myeloma cells with the spleen cells from mouse immunized with PA and LF [15,52]. All experiments were approved by the Animal Care and Use Committee of the Beijing Institute of Biotechnology (identification code: 20140611; date of approval: 11 June 2014). The epitope of the anti-PA mAb is the PA domain 4 (596–735), and the epitope of the anti-LF mAb is the LF central region (276–576). HRP-labeled goat anti-mouse IgG was purchased from Santa Cruz Biotechnology (Santa Cruz, CA, USA). Trypsin and protease inhibitors cocktail were purchased from Sigma-Aldrich (Steinheim, Germany). All other reagents were purchased from Sigma-Aldrich unless otherwise specified.

### 4.2. Bacterial Strain

Two attenuated vaccine strains A16R (pXO1+, pXO2−) and Sterne (pXO1+, pXO2−) were used in this study. A16R is the only human anthrax vaccine in China, which was derived from the wild strain A16 that lost the capsule-coding plasmid pXO2 [2]. The Sterne strain is also a non-capsulated, toxin-producing strain and is currently commonly used as an animal anthrax vaccine [2]. The A16R strain was provided by the Lanzhou Institute of Biological Products (Lanzhou, China)., and the Sterne strain was provided by the Qinghai Biological Medicine Factory (Xining, China).

### 4.3. Cultivation and Sampling

A16R and Sterne strains were cultivated as described previously [53]. In brief, clonal cells were inoculated into a tube with 5 mL Luria-Bertani (LB) liquid medium at 37°C with vigorous agitation for 12 h. From this culture, 50 μL was used to inoculate another tube with 5 mL LB medium for 12 h. Erlenmeyer flasks each containing 100 mL (total 500 mL) were sterilized by autoclaving at 121°C for 15 min and then warmed to 37 °C prior to inoculation with 1 mL of each culture. In the experiments to measure the LT production rate with protease inhibitors, protease inhibitors cocktail (final concentration of 10 μL/mL) was added in the LB medium. The bottles were incubated at 37 °C with vigorous agitation for up to 28 h. Culture supernatant samples were taken throughout the time course and sterilized by passing through a 0.22-µm filter. After hyperfiltration using a 5 mL centrifugal filter with high sample recovery (>90%), a fraction of the culture supernatant sample was concentrated to 10×. All samples were stored frozen (–80 °C) in aliquots until analysis.

### 4.4. Typical Growth Profile and Viable Cell Counts

The OD_600_ of each culture was determined using an ultraviolet photometer (Unico-7200, UNICO, Dayton, NJ, USA) throughout the time course. Viable counts were also obtained by plating serial 10-fold dilutions (100 μL aliquots) onto LB agar plates in duplicate and incubating at 37 °C for 48 h. Plates with 30–200 colonies were used to calculate the viable counts of the sample [35].

### 4.5. Western Blot Analysis of PA and LF

Electrophoresis of supernatant samples was performed using 12% SDS polyacrylamide gels, with 20 μL of culture supernatant loaded for each sample. The immunoblotting conditions used were the same as those described previously [49]. Briefly, for immunological detection, the SDS-PAGE gels were blotted onto the nitrocellulose membranes, which were then probed with specific primary antibodies and peroxidase-conjugated secondary antibodies with chemiluminescent substrates. Protein bands were probed with 1:10,000 diluted HRP-conjugated goat anti-rabbit IgG and washed four times as described above. Chemiluminescence was applied as instructed by the manufacturer.

### 4.6. Sandwich ELISA

Concentrations of PA and LF in samples of filtered culture supernatants were assessed using an ELISA as described previously [15]. ELISA plates (96-well) were coated with a rabbit pAb specific for PA (or LF) (100 μL, final concentration of 2 μg/mL) in coating buffer overnight at 4 °C. After washing three times using PBST, the plates were incubated with blanking buffer (100 μL, PBST and 2% (*w*/*v*) BSA) for 1 h at 37 °C with continuous shaking. Thereafter, culture supernatant samples containing PA (or LF) were serially diluted in blanking buffer and added to the plates at 100 μL per well. After 1 h at 37 °C with continuous shaking, the plates were washed as above. HRP-labeled anti-PA (or LF) mAbs were then added to the plates for incubation at 37 °C for 1 h with continuous shaking, followed by three washes with PBST. After adding the substrate solution, the plates were again incubated at 37 °C with continuous shaking for 25–30 min. The reaction was then stopped with 2 mol/L sulfuric acid (50 μL per well), and the absorbance measured at 450/630 nm using a microplate reader (Bio-Rad, Hercules, CA, USA).

In the co-IP assay, an anti-PA pAb (100 µL, final concentration of 2 μg/mL) was added to ELISA plates for capturing PA. The samples added to plates coated by anti-PA pAbs was 1:10 diluted. An anti-LF mAb (100 µL, final concentration of 2 μg/mL) was then added to detect LF as described above. The reverse co-IP assay was performed as well, in which an anti-LF pAb was used to pull down LF, and an anti-PA mAb was used for detection as described above. The samples added to plates coated by anti-LF pAbs was 1:1 diluted. The rPA and rLF proteins at fixed amounts (50 ng) and LT heptamers complexes were also detected in the co-IP assay as a control. The LT heptamers complexes were prepared as described previously [21]. PA83 was treated with trypsin at a final trypsin/PA weight ratio of 1:1000 for 30 min at room temperature. The reaction was stopped by adding a 10-fold excess of trypsin inhibitor. PA83 proteins were cleaved to PA63, the latter would then spontaneously form heptamers and in turn recruit LF (final concentration of 50μg/mL), resulting in pre-formed LT heptamers complexes.

### 4.7. Cytotoxicity Assay

The mouse macrophage cell line J774 has been found to be uniquely sensitive to LT. These cells can be killed by LF in as little as 90 min and therefore can be used in detection of LT lethal activity. The macrophage lysis assay was established essentially as described before with slight modifications [15]. Optimal assay conditions were determined experimentally. Mouse macrophage J774A.1 cells were plated at 3 × 10^5^ cells/well in 96-well plates and cultured for 24 h before treatment. The growth medium was then removed by cautious aspiration and replaced with 100 μL of new medium containing culture supernatant samples (diluted 1:10). Cell viability was assayed 4 h after treatment by replacing the medium with 100 μL of medium (MEM plus 2% FBS) containing 1 mg/mL MTT. After 1 h of incubation at 37 °C, the medium was removed, and the blue pigment produced by the viable cells was dissolved in 50 μL/well of 0.5% (w/v) SDS and 25 mM hydrochloric acid in 90% (*v*/*v*) isopropanol. The plates were then vortexed, and oxidized MTT was measured as the absorbance at 570 nm using a Model 550 microplate reader (Bio-Rad, Hercules, CA, USA). Cell viability was calculated as a percentage using the equation (OD sample – OD death control)/(OD live control – OD death control), where “live control” wells contained 1 μg/mL PA alone and “death control” wells contained both 1 μg/mL PA and 100 ng/mL LF. EC_50_ values were determined by nonlinear regression sigmoidal dose-response analysis with variable slopes. Each assay was performed at least three times, with duplicates within each assay.

### 4.8. Cytotoxicity-Based Method for Detection of PA and LF

Concentrations of active PA and LF in samples of filtered culture supernatants were assessed using a cytotoxicity-based method. With a fixed amount of excess PA (or LF), the change in cytotoxicity can be attributed to the varying amount of LF (or PA). We used two of the same samples to detect active PA and LF individually. LF activity in samples was assessed based on the ability together with PA to kill J774A.1 cells *in vitro*. As described above, PA (1 μg/mL) and varying amounts of 10× culture supernatant samples were applied, and cell viability was then assessed 4 h later by the MTT assay. As the concentration of PA was in excess, the decisive factor was the concentration of LF, which could be obtained based on the cell viability. Thus, a cytotoxicity-based method for detection of active LF was developed [43]. A similar cytotoxicity-based method for detection of active PA was also established. The growth medium was removed by carefully aspirating and replacing it with 100 μL of new medium (50 ng/mL LF) containing 10× culture supernatant samples. Cell viability was then assessed as above.

### 4.9. Data Analysis

Unless otherwise specified, results are expressed as the mean ± SEM for the values obtained from multiple experiments. Analyses were performed using GraphPad Prism 5 (GraphPad Software, La Jolla, CA, USA). Statistical significance was determined at * *p* ≤ 0.05, ** *p* ≤ 0.01, and *** *p* ≤ 0.001.

## Figures and Tables

**Table 1:** Absorbance readings (OD) for detection of LT heptamers in culture supernatants of A16R strain by co-IP assay.

Sample	Absorbance Readings (OD)	
Capture	anti-PA pAb	anti-LF pAb
Detect	anti-LF mAb	anti-PA mAb
Negative Control	0.108 ± 0.014	0.035 ± 0.006
LT complexes	0.820 ± 0.071	0.718 ± 0.059
rPA (5 ng)	0.101 ± 0.012	0.027 ± 0.005
rLF (50 ng)	0.109 ± 0.015	0.035 ± 0.006
4 h	0.098 ± 0.017	0.026 ± 0.006
8 h	0.112 ± 0.016	0.027 ± 0.006
12 h	0.111 ± 0.021	0.028 ± 0.008
16 h	0.102 ± 0.011	0.026 ± 0.010
20 h	0.105 ± 0.015	0.024 ± 0.006
24 h	0.09 ± 0.012	0.032 ± 0.007

Average OD values shown with standard deviation (SD) were derived from three readings. Samples added to plates coated with anti-PA pAbs were 1:10 diluted, while samples added to plates coated with anti-LF pAbs were 1:1 diluted. The label OD means OD < cut-off level (CO) (CO = negative control average + 3 SD).
